# Urinary Levels of Titin-N Fragment, a Skeletal Muscle Damage Marker, are Increased in Subjects with Nonalcoholic Fatty Liver Disease

**DOI:** 10.1038/s41598-019-56121-7

**Published:** 2019-12-20

**Authors:** Natsumi Oshida, Takashi Shida, Sechang Oh, Taeho Kim, Tomonori Isobe, Yoshikazu Okamoto, Takashi Kamimaki, Kosuke Okada, Hideo Suzuki, Shun-ichi Ariizumi, Masakazu Yamamoto, Junichi Shoda

**Affiliations:** 1 Division of Laboratory Medicine, Tsukuba University Hospital, Tsukuba, Ibaraki 305-8576 Japan; 2 Tsukuba Preventive Medicine Research Center, Tsukuba University Hospital, Tsukuba, Ibaraki 305-8576 Japan; 3 Tsukuba Sports Medicine and Health Science Center, Tsukuba University Hospital, Tsukuba, Ibaraki 305-8576 Japan; 4 Medical Sciences, Faculty of Medicine, University of Tsukuba, Tsukuba, Ibaraki 305-8575 Japan; 5 Division of Radiology, Faculty of Medicine, University of Tsukuba, Tsukuba, Ibaraki 305-8575 Japan; 6 Institute of Gastroenterology Surgery, Tokyo Women’s Medical University, Tokyo, 162-8666 Japan

**Keywords:** Biochemistry, Medical research

## Abstract

Sarcopenia is a pathological condition affecting the development and progression of NAFLD. Urinary levels of titin-N fragment, a biomarker reflecting muscle damage, were measured in NAFLD subjects, and analyzed in a retrospective manner for possible correlations with NAFLD pathophysiology to assess their clinical relevance. This study enrolled 153 NAFLD subjects and 100 subjects without NAFLD, obesity or diabetes mellitus (non-NAFLD). NAFLD subjects had more decreased knee extension strength. NAFLD subjects had greater subcutaneous fat thickness and echo intensity (brightness) of the rectus femoris muscle on ultrasound images; higher levels of the intra- and extra-myocellular lipids (IMCL, EMCL) using ^1^H-MRS. Urinary titin-N fragment levels were increased with increasing age but not different between males and females. NAFLD subjects had higher titin-N fragment levels than non-NAFLD subjects. The levels were negatively correlated with skeletal muscle mass and knee extension strength and positively correlated with muscle echo intensity, EMCL, and liver fibrosis scores (NAFLD fibrosis score, FIB-4 index). Multivariate analysis revealed that factors affecting the levels were skeletal mass index, leg skeletal muscle mass, liver stiffness, and NAFLD fibrosis score. Urinary levels of titin-N fragment reflected skeletal muscle deterioration and functional decline, and was closely associated with hepatic pathological conditions in NAFLD subjects.

## Introduction

Recent Japanese data on medical checkups show that the prevalence of adults with abnormal liver function has been rapidly increasing^1^. A major reason behind this is an increase in the incidence of nonalcoholic fatty liver disease (NAFLD) caused by visceral obesity. NAFLD is caused by a variety of damaging factors that induce complex crosstalk and pathological interactions between the liver and other organs, according to the multiple parallel hit hypothesis^2^, an accurate explanation of NAFLD pathogenesis.

Interestingly, a relationship between decreased skeletal muscle mass and life prognosis in chronic liver disease has recently been reported, with the following findings: decreased exercise tolerance due to decreased muscle mass is associated with shortened life prognosis in liver cirrhosis^3^; increased serum levels of myostatin, a myokine negatively regulating muscle protein synthesis, is correlated with a muscle mass loss in liver cirrhosis patients and associated with the reduced overall survival rate^4^; decreased muscle mass is a poor independent prognostic factor for hepatocellular carcinoma^5,6^; and decreased muscle mass increases the risk of developing NAFLD^7–9^ and liver fibrosis in NAFLD patients^9^. On the other hand, in a longitudinal study, increases in muscle mass over time were associated with benefits either in the development of NAFLD or the improvement of NAFLD pathological conditions^10^.

We have also shown that abnormal body composition consisting of increased visceral fat mass and decreased skeletal muscle mass has an impact on the progression of NAFLD^11^. Furthermore, liver fibrosis was found to be worsened in NAFLD subjects whose clinical course could be followed and whose skeletal muscle mass to visceral fat area ratio (SV ratio) was decreased at the time of re-examination compared with at the time of initial examination^12^.

Sarcopenia is characterized by progressive and systemic loss of skeletal muscle mass and decreased muscle strength^13^. Recent studies have demonstrated that common factors such as insulin resistance, vitamin D deficiency, chronic inflammation, myokines, cytokines, and decreased physical activity are involved in the pathogenesis of both sarcopenia and NAFLD, and play specific roles, in these diseases, with overlapping pathophysiology and circulatory associations^14^.

Intramuscular and intermuscular fat accumulates when muscle cells are decreased in sarcopenic skeletal muscles. This is called muscle steatosis. Mortality from chronic liver diseases has been reported to be 1.5 to 2 times higher in patients with muscle steatosis than in those without it^15^. Quantitative and qualitative assessment of skeletal muscles is important in the management of chronic liver diseases because muscle steatosis, in addition to the loss of skeletal muscles, is a poor prognostic factor for chronic liver diseases^6^.

In recent years, ultrasonographic assessment of skeletal muscles has attracted attention. Muscle thickness measured with an ultrasonic device is strongly correlated with measurement using computed tomography (CT) and magnetic resonance imaging (MRI), and its validity and reproducibility as a muscle mass parameter have been demonstrated^16^. On ultrasound imaging, muscle echo intensity is increased in skeletal muscles with increased non-contractile tissue because interface with different acoustic impedance increase as the non-contractile tissue increases. In fact, ultrasonographic images of skeletal muscles in the elderly reportedly show greater echo intensity than those in younger individuals, as well as qualitative changes; e.g. an increase in the proportion of non-contractile tissues, including connective tissues in skeletal muscles^17^; and muscle echo intensity is associated with fatty infiltration on muscle biopsy^18^.

Enzyme-linked immunosorbent assay (ELISA) of titin-N fragment has recently been established as a biomarker reflecting muscle damages^19^. Titin is decomposed by proteolytic enzymes such as calpain if muscles are damaged by muscle diseases or vigorous exercise, and various titin fragments are detected in serum and urine. In particular, N- and C-terminal fragments of titin are most commonly detected, and urinary levels of titin-N fragment are reportedly approximately 700 times higher than normal in patients with Duchenne muscular dystrophy^20^. Thus, urinary levels of titin-N fragment are expected to serve as a noninvasive biomarker, reflecting the muscle catabolic states in NAFLD.

In this study, to predict pathological conditions in skeletal muscles through liver-muscle crosstalk, we analyzed ultrasound images of skeletal muscles, muscle mass, muscle strength, sarcopenia index, and liver pathophysiological factors. Furthermore, urinary levels of titin-N fragment, a biomarker reflecting muscle damages, were measured in NAFLD subjects. The clinical relevance of titin-N fragment was assessed to discuss its possible correlations with clinical data on the muscle and liver items.

## Results

### Comparison of the prevalence rates of lifestyle-related diseases of study subjects

The frequencies of obesity in the young-age (≤30 years), middle-age (31–60 years), and old-age (≥61 years) groups of NAFLD subjects were 71.4%, 77.5%, and 58.8%, respectively (Table 1). The frequency of obesity was lower in the old-age group than in the young-age and middle-age groups. In all age groups, the morbidities of lifestyle-related diseases, such as type 2 diabetes mellitus, dyslipidemia, and hypertension, were higher in NAFLD subjects than in non-NAFLD subjects.

**Table 1:** Comparisons of the gender and prevalence rates of life style-related diseases for study subjects in younger, middle, and elder age groups.

Parameter	≤30		31–60		≥61		*P* value *Chi*-square test
	NAFLD	non-NAFLD	NAFLD	non-NAFLD	NAFLD	non-NAFLD	
	(n = 14)	(n = 41)	(n = 71)	(n = 35)	(n = 68)	(n = 24)	
**Gender, % male**	75.0	127.8	109.8	59.1	78.9	60.0	0.316
**Life style-related diseases**							
Obesity (BMI ≥ 25) (%)	71.4	0	77.5	0	58.8	0	<0,001
Diabetes mellitus (%)	28.6	0	25.4	0	48.5	0	<0,001
Dyslipidemia (%)	30.8	15.0	55.7	8.8	51.6	22.7	<0,001
Hypertension (%)	50.0	0	40.7	12.0	65.5	35.0	<0,001

Categorical variables were analyzed by using the *chi-*square test.

### Comparison of anthropometric characteristics of study subjects

BMI, visceral fat area (VFA), and skeletal muscle mass were higher in NAFLD subjects than in non-NAFLD subjects in the young-age (≤30 years), middle-age (31–60 years), and old-age (≥61 years) groups (Table 2). Body mass index (BMI) decreased with age in NAFLD subjects. VFA increased with age in non-NAFLD subjects. Skeletal muscle mass decreased with age in NAFLD and non-NAFLD subjects.

**Table 2:** Comparisons of the prevalence rates of life style-related diseases, anthropometric characteristics, hepatic abnormalities, insulin resistance and lipid profiles, and markers of fibrosis for study subjects in younger, middle, and elder age groups.

Parameter	≤30		*P* value	31–60		*P* value	≥61		*P* value	*P* value					
	NAFLD	non-NAFLD		NAFLD	non-NAFLD		NAFLD	non-NAFLD		NAFLD			non-NAFLD		
	(N, n = 14)	(nN, n = 41)	N vs. nN	(n = 71)	(n = 35)	N vs. nN	(n = 68)	(n = 24)	N vs. nN	≤30 vs. 31–60	≤30 vs. ≥61	31–60 vs. ≥61	≤30 vs. 31–60	≤30 vs. ≥61	31–60 vs. ≥61
**Anthropometric characteristics**															
Weight, kg	89.1 ± 7.5	58.0 ± 1.4	<0.001	78.2 ± 2.1	55.6 ± 1.5	<0.001	66.0 ± 1.3	52.4 ± 1.3	<0.001	0.070	<0.001	<0.001	0.450	<0.05	0.315
Body mass index, kg·m^−2^	32.6 ± 2.3	21.0 ± 0.4	<0.001	28.5 ± 0.6	20.8 ± 0.3	<0.001	26.2 ± 0.5	20.9 ± 0.5	<0.001	<0.05	<0.001	<0.05	0.943	0.993	0.983
Skeletal muscle mass, kg	29.3 ± 2.4	26.2 ± 0.8	0.128	28.4 ± 0.8	23.8 ± 0.9	<0.001	23.7 ± 0.5	21.6 ± 0.6	<0.05	0.877	<0.01	<0.001	0.086	<0.001	0.187
Fat mass, kg	36.6 ± 4.6	10.9 ± 0.5	<0.001	27.5 ± 1.3	12.1 ± 0.6	<0.001	22.3 ± 1.0	12.1 ± 0.9	<0.001	<0.05	<0.001	<0.05	0.349	0.403	1.000
Fat-free mass, kg	52.5 ± 4.0	47.1 ± 1.3	0.108	51.2 ± 1.3	43.6 ± 1.4	<0.001	8.72 ± 1.0	43.7 ± 0.9	<0.05	0.908	<0.05	<0.001	0.132	<0.01	0.270
Visceral fat area, cm^3^	135.4 ± 17.2	40.5 ± 2.8	<0.001	123.9 ± 4.4	65.2 ± 3.5	<0.001	119.4 ± 4.4	85.6 ± 5.4	<0.001	0.591	0.369	0.782	<0.001	<0.001	<0.001
**Hepatic abnormalities**															
CAP, dB·m^−1^	314.2 ± 63.7	167.2 ± 28.6	<0.001	292.6 ± 51.0	186.9 ± 36.9	<0.001	268.7 ± 42.6	179.5 ± 28.2	<0.001	0.309	<0.01	<0.05	<0.05	0.280	0.640
LS, kPa	9.4 ± 6.5	4.5 ± 0.8	<0.001	8.1 ± 5.3	4.5 ± 0.9	<0.001	8.4 ± 8.3	4.4 ± 0.7	<0.05	0.810	0.878	0.971	0.997	0.981	0.993
Albumin, g·dL^−1^	4.4 ± 0.10	4.6 ± 0.04	<0.05	4.5 ± 0.03	4.4 ± 0.05	0.408	4.3 ± 0.03	4.4 ± 0.04	0.489	0.850	0.357	<0.01	<0.01	<0.001	0.523
AST, U·L^−1^	42.5 ± 7.6	20.6 ± 1.0	<0.001	35.5 ± 2.6	22.1 ± 1.1	<0.001	31.8 ± 2.1	23.4 ± 0.9	<0.05	0.504	0.215	0.570	0.544	0.162	0.667
ALT, U·L^−1^	71.2 ± 15.6	18.5 ± 1.5	<0.001	49.1 ± 4.5	18.3 ± 1.1	<0.001	33.2 ± 2.2	16.5 ± 0.8	<0.001	0.073	<0.001	<0.05	0.996	0.550	0.618
γ-GT, U·L^−1^	42.3 ± 7.0	26.9 ± 4.0	0.059	62.8 ± 6.6	30.8 ± 6.3	<0.01	61.0 ± 8.6	28.1 ± 7.3	<0.05	0.510	0.612	0.958	0.862	0.989	0.945
**Insulin resistance and lipid profiles**															
FPG, mg·dL^−1^	102.2 ± 6.5	87.7 ± 1.0	<0.001	112.8 ± 3.2	90.2 ± 1.2	<0.001	126.6 ± 4.0	97.2 ± 1.8	<0.001	0.438	<0.05	<0.05	0.296	<0.001	<0.01
Insulin, μU·mL^−1^	23.7 ± 5.0	6.4 ± 0.5	<0.001	16.4 ± 2.2	4.8 ± 0.5	<0.001	13.0 ± 1.9	6.7 ± 1.7	<0.001	0.330	0.103	0.506	0.215	0.952	0.242
HOMA-IR	6.4 ± 1.5	1.4 ± 0.1	<0.001	4.5 ± 0.7	1.1 ± 0.1	<0.001	4.1 ± 0.8	1.8 ± 0.5	0.149	0.512	0.408	0.947	0.414	0.383	0.068
HbA1c, %	6.2 ± 0.36	5.2 ± 0.03	<0.001	6.0 ± 0.14	5.3 ± 0.06	<0.001	6.6 ± 0.13	5.6 ± 0.08	<0.001	0.871	0.475	<0.05	0.221	<0.001	<0.001
HDL-C, mg·dL^−1^	53.2 ± 4.3	66.4 ± 2.3	<0.01	50.9 ± 1.5	69.1 ± 2.8	<0.001	51.2 ± 1.6	77.0 ± 5.1	<0.001	0.823	0.845	0.998	0.737	0.079	0.274
LDL-C, mg·dL^−1^	130.3 ± 9.7	102.2 ± 4.7	<0.01	125.1 ± 3.5	111.9 ± 3.8	<0.05	119.1 ± 4.0	119.7 ± 8.7	0.951	0.841	0.464	0.512	0.320	0.117	0.666
TG, mg·dL^−1^	106.8 ± 10.2	70.8 ± 5.1	<0.01	137.7 ± 9.2	83.4 ± 13.0	<0.001	133.5 ± 9.1	74.3 ± 8.0	<0.001	0.340	0.461	0.929	0.565	0.965	0.801
**Markers of fibrosis**															
Hyaluronic acid, ng·mL^−1^	15.7 ± 2.2	19.7 ± 1.8	0.235	39.1 ± 6.4	20.5 ± 2.6	<0.05	76.9 ± 12.6	56.6 ± 8.9	0.446	0.570	<0.05	<0.05	0.984	<0.001	<0.001
Type IV collagen, ng·mL^−1^	118.2 ± 8.8	116.5 ± 4.1	0.854	135.5 ± 5.0	113.3 ± 4.0	<0.01	141.2 ± 4.8	129.3 ± 6.1	0.263	0.326	0.129	0.625	0.844	0.198	0.094
Platelet, × 10^9^·L^−1^	278.2 ± 11.4	258.6 ± 8.4	0.231	233.2 ± 7.7	259.8 ± 11.6	0.054	205.6 ± 7.4	217.5 ± 9.0	0.377	<0.05	<0.001	<0.05	0.996	<0.05	<0.05
NAFLD fibrosis score	−3.08 ± 0.30	−3.92 ± 0.12	<0.01	−2.09 ± 0.15	−3.09 ± 0.1	<0.001	−0.65 ± 0.16	−1.44 ± 0.17	<0.01	<0.05	<0.001	<0.001	<0.001	<0.001	<0.001
FIB-4 index	0.48 ± 0.06	0.48 ± 0.02	0.940	1.25 ± 0.11	0.95 ± 0.06	0.075	2.02 ± 0.12	1.91 ± 0.10	0.602	<0.05	<0.001	<0.001	<0.001	<0.001	<0.001
**Other biochemical characteristics**															
hs-CRP (mg/dL)	0.48 ± 0.17	0.08 ± 0.02	<0.001	0.19 ± 0.02	0.03 ± 0.03	<0.01	0.16 ± 0.02	0.08 ± 0.02	<0.05	<0.001	<0.001	0.680	0.956	0.987	0.994
Ferritin (mg/mL)	71.9 ± 13.7	60.7 ± 7.4	0.471	146.6 ± 24.1	67.9 ± 12.7	<0.05	125.8 ± 12.9	72.4 ± 13.3	0.053	0.272	0.499	0.730	0.860	0.770	0.965
TBARS (µmoles/L)	3.87 ± 0.61	4.51 ± 1.08	0.733	4.11 ± 0.52	3.02 ± 0.52	0.216	4.47 ± 0.74	2.96 ± 0.45	0.272	0.985	0.914	0.909	0.423	0.485	0.999
Myostatin (ng/mL)	4.6 ± 1.0	3.9 ± 0.7	0.612	8.6 ± 0.7	5.2 ± 1.2	<0.05	7.6 ± 0.6	6.9 ± 1.1	0.559	<0.05	0.149	0.597	0.616	0.115	0.523
IGF-1 (ng/mL)	3.02 ± 0.24	1.81 ± 0.08	<0.001	2.48 ± 0.13	1.36 ± 0.07	<0.001	2.21 ± 0.11	1.92 ± 0.26	0.238	0.156	<0.05	0.501	<0.05	<0.05	<0.05
DHEA (ng/mL)	29.2 ± 7.8	37.0 ± 2.8	0.246	21.6 ± 2.5	24.2 ± 2.6	0.531	12.2 ± 1.2	9.6 ± 1.6	0.238	0.315	<0.01	<0.001	<0.01	<0.001	<0.001
DHT (pg/mL)	523.3 ± 178.5	671.3 ± 181.3	0.639	385.2 ± 65.3	452.9 ± 153.3	0.633	287.0 ± 60.4	175.3 ± 30.9	0.310	0.660	0.285	0.501	0.562	0.086	0.485
Vitamin D (ng/mL)	32.6 ± 6.7	55.1 ± 3.6	<0.01	43.0 ± 2.9	53.2 ± 5.8	0.088	63.1 ± 4.6	58.9 ± 5.7	0.628	0.468	<0.05	<0.001	0.955	0.858	0.733

Values are presented as the means ± SE. To compare between groups, all dependent variables were analyzed by using ANCOVA with adjustment for gender as a covariate.

**Figure 1:**
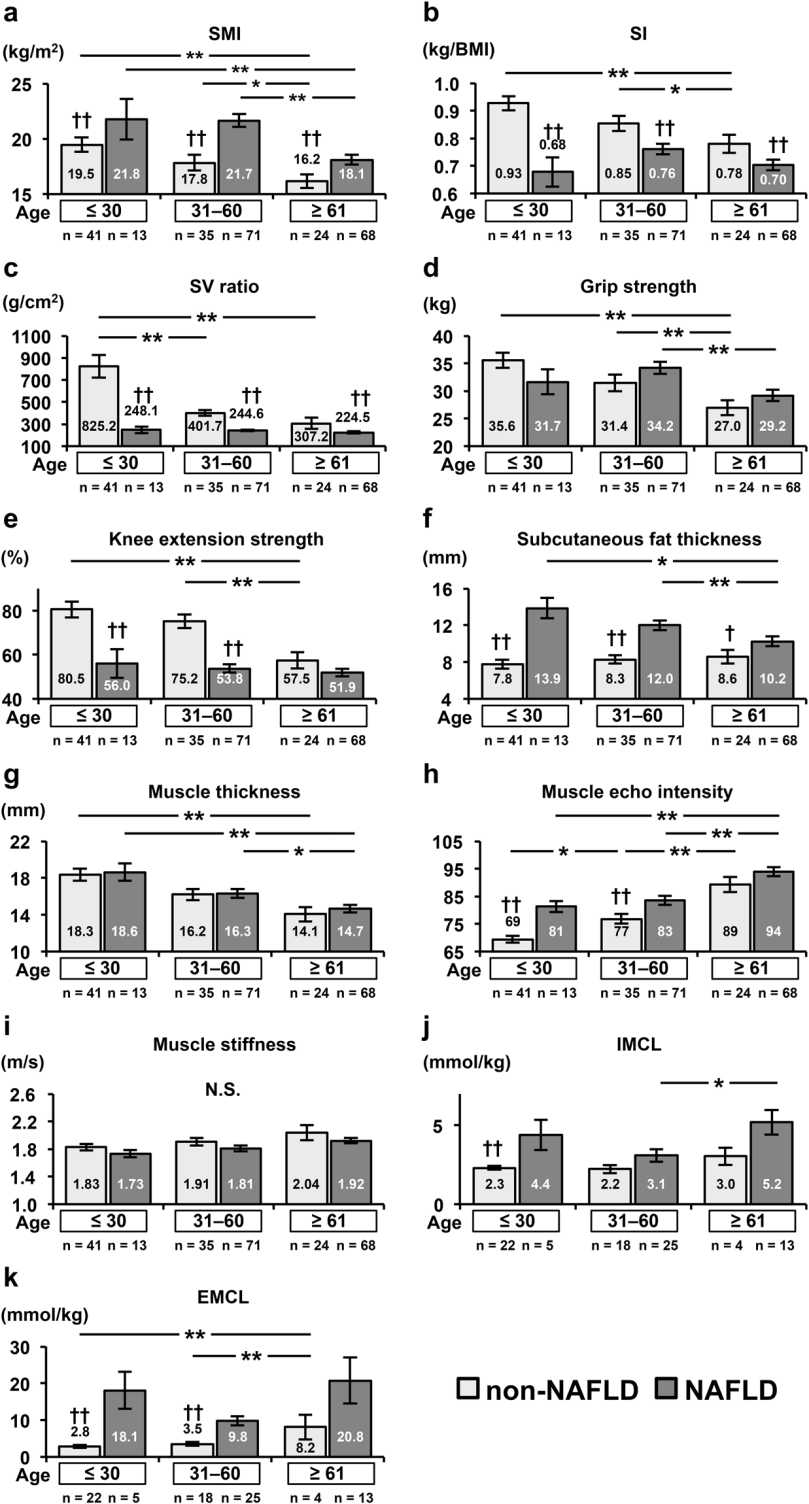
The comparisons of skeletal muscle index (SMI) (**a**), sarcopenia index (SI) (**b**), skeletal muscle mass to visceral fat area ratio (SV ratio) (**c**), grip strength (**d**), knee extension strength (**e**), subcutaneous fat thickness over the thigh (**f**), thickness (**g**), elasticity (**h**), echo intensity of the rectus femoris muscle **(i**), intramyocellular lipid (IMCL) (**j**), and extramyocellular lipid (EMCL) of quadriceps femoris muscle (**k**) in NAFLD subjects and non-NAFLD subjects. The subjects were classified into 3 groups by age (≤30 years, 31 to 60 years, ≥61 years); these 3 groups were compared using an ANCOVA with adjustment for gender as a covariate. *P < 0.05, **P < 0.01; significantly different between the age groups. ^††^*P* < 0.01; significantly different between the NAFLD and non-NAFLD subjects.

Skeletal mass index (SMI) was higher in NAFLD subjects than in non-NAFLD subjects in all age groups; SMI decreased with age in NAFLD and non-NAFLD subjects (Fig. 1). In contrast, sarcopenia index (SI) and SV ratios were lower in NAFLD subjects than in non-NAFLD subjects in all age groups, and SI and SV ratios decreased with age in non-NAFLD subjects.

### Comparison of NAFLD-related hepatic conditions of study subjects

Aspartate aminotransferase (AST) and alanine aminotransferase (ALT) levels were higher in NAFLD subjects than in non-NAFLD subjects in all age groups (Table 2). ALT levels decreased with age in subjects with NAFLD. Furthermore, gamma-glutamyl transpeptidase (γGT) levels were higher in NAFLD subjects than in non-NAFLD subjects in all age groups. Levels of hyaluronic acid (HA) and type IV collagen as liver fibrosis markers were higher in NAFLD subjects than in non-NAFLD subjects only in the middle-age group. HA levels increased with age in NAFLD and non-NAFLD subjects.

NAFLD fibrosis score (NFS), a predictor of fibrosis, was higher in NAFLD subjects than in non-NAFLD subjects in all age groups. Fibrosis 4 (FIB-4) index did not differ between NAFLD and non-NAFLD subjects in all age groups. Both NFS and FIB-4 index increased with age in NAFLD and non-NAFLD subjects.

On transient elastography, liver stiffness measurement (LSM) and controlled attenuation parameter (CAP) were higher in NAFLD subjects than in non-NAFLD subjects in all age groups. LSM did not change with age in NAFLD and non-NAFLD subjects. CAP decreased with age in NAFLD subjects.

### Comparison of skeletal muscle-related characteristics of study subjects

Grip strength did not differ between NAFLD and non-NAFLD subjects in all age groups, and grip strength decreased with age in non-NAFLD subjects (Fig. 1). Knee extension strength was lower in NAFLD subjects than in non-NAFLD subjects in the young-age and middle-age groups; knee extension strength decreased with age in non-NAFLD subjects.

**Figure 2:**
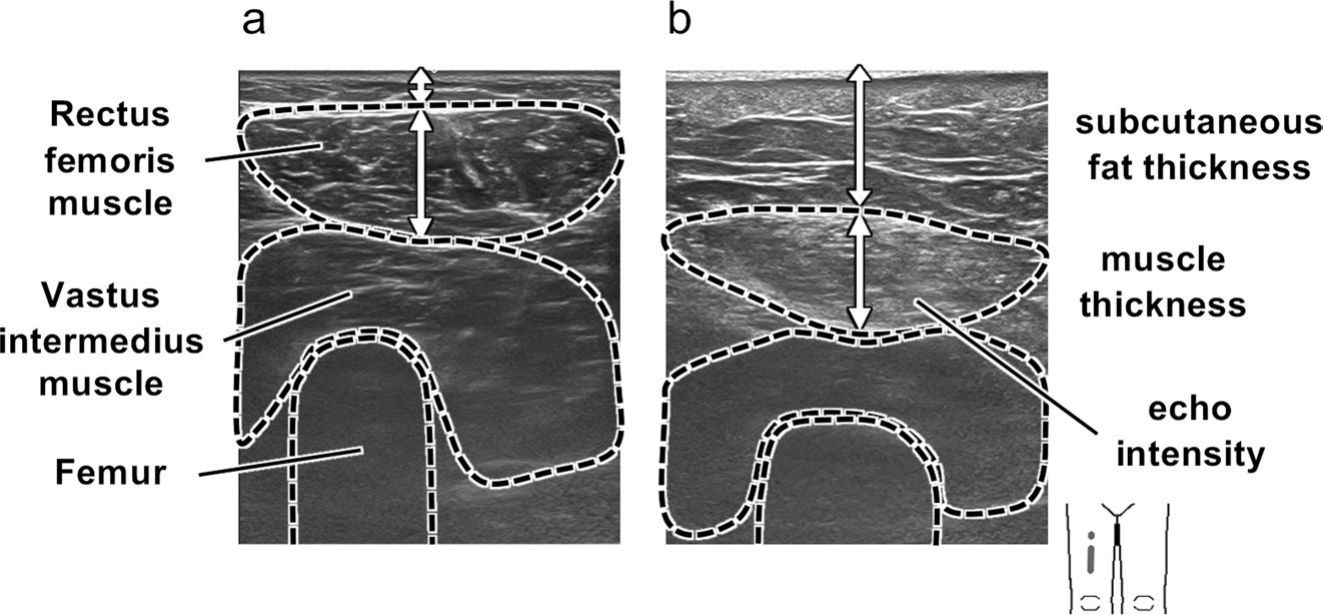
Representative ultrasound images of the rectus femoris muscle in a non-NAFLD female subject (**a**) and that in a NAFLD female subject (**b**). The NAFLD subject had greater subcutaneous fat thickness and higher echo intensity of the rectus femoris muscle on the images.

Figure 2 shows representative ultrasound images of the rectus femoris muscles of NAFLD and non-NAFLD subjects. As shown in Fig. 1, subcutaneous fat was thicker in NAFLD subjects than in non-NAFLD subjects in all age groups, and the thickness of subcutaneous fat decreased with age in NAFLD subjects. Muscle thickness did not differ between NAFLD and non-NAFLD subjects in all age groups, and muscle thickness decreased with age in NAFLD and non-NAFLD subjects. Muscle echo intensity was higher in NAFLD subjects than in non-NAFLD subjects in the young-age and middle-age groups, and muscle echo intensity increased with age in NAFLD and non-NAFLD subjects. Muscle elasticity did not differ between NAFLD and non-NAFLD subjects in all age groups and also did not change with age.

On proton magnetic resonance spectroscopy (^1^H-MRS) of skeletal muscle, intramyocellular lipid (IMCL) content was higher in NAFLD subjects than in non-NAFLD subjects in the young-age group; IMCL content did not change with age in NAFLD and non-NAFLD subjects. Extramyocellular lipid (EMCL) content was higher in NAFLD subjects than in non-NAFLD subjects in the young-age and middle-age groups; EMCL content did not change with age in NAFLD subjects but increased with age in non-NAFLD subjects.

Muscle echo intensity on ultrasound showed negative correlations with grip strength (r = −0.343; *P* < 0.001) and knee extension strength (r = −0.397; *P* < 0.001), whereas it showed positive correlations with IMCL (r = 0.285; *P* < 0.01) and EMCL (r = 0.565; *P* < 0.001) content; a particularly stronger correlation in relation to the other coefficients was observed between muscle echo intensity and EMCL content. Furthermore, a positive correlation was observed between NFS, a predictor of liver fibrosis (r = 0.513; *P* < 0.001) and FIB-4 index (r = 0.500, *P* < 0.001).

### Comparison of insulin resistance status and dyslipidemia of study subjects

Fasting plasma glucose (FPG) levels, fasting insulin levels, and homeostasis model assessment-insulin resistance (HOMA-IR) were higher in NAFLD subjects than in non-NAFLD subjects in the young-age and middle-age groups. FPG levels increased with age in NAFLD and non-NAFLD subjects (Table 2).

In terms of serum lipids, low-density lipoprotein cholesterol (LDL-C) levels were higher in NAFLD subjects than in non-NAFLD subjects in the young-age and middle-age groups. Triglyceride (TG) levels were higher in NAFLD subjects than in non-NAFLD subjects in all age groups. Contrastingly, high-density lipoprotein cholesterol (HDL-C) levels were lower in NAFLD subjects than in non-NAFLD subjects in all age groups. LDL-C, TG, and HDL-C levels did not change with age in NAFLD and non-NAFLD subjects.

### Comparison of biochemical characteristics of study subjects

Levels of inflammatory and oxidative stress markers such as high-sensitivity C-reactive protein (hs-CRP), ferritin, and 2-thiobarbituric acid reactive substances (TBARS) were measured. Hs-CRP levels were higher in NAFLD subjects than in non-NAFLD subjects in all age groups, and hs-CRP levels decreased with age in NAFLD subjects (Table 2). Ferritin levels were higher in NAFLD subjects than in non-NAFLD subjects in the middle-age group, and ferritin levels did not change with age. The level of TBARS, an oxidative stress marker, did not differ between NAFLD and non-NAFLD subjects in all age groups or also did not change with age.

Levels of myostatin, which is associated with muscle atrophy and liver fibrosis, were higher in NAFLD subjects than in non-NAFLD subjects in the middle-age group. Factors associated with muscle synthesis, such as insulin-like growth factor-1 (IGF-1), dehydroepiandrosterone (DHEA), dihydrotestosterone, and vitamin D, were measured. IGF-1 levels were higher in NAFLD subjects than in non-NAFLD subjects in the young-age and middle-age groups, and IGF-1 levels decreased with age in NAFLD subjects. DHEA levels did not differ between NAFLD and non-NAFLD subjects but decreased with age in NAFLD and non-NAFLD subjects. Vitamin D levels were lower in NAFLD subjects than in non-NAFLD subjects in the young-age group, and vitamin D levels increased with age in NAFLD subjects.

### Comparison of titin N-fragment levels in the urine of study subjects

**Figure 3:**
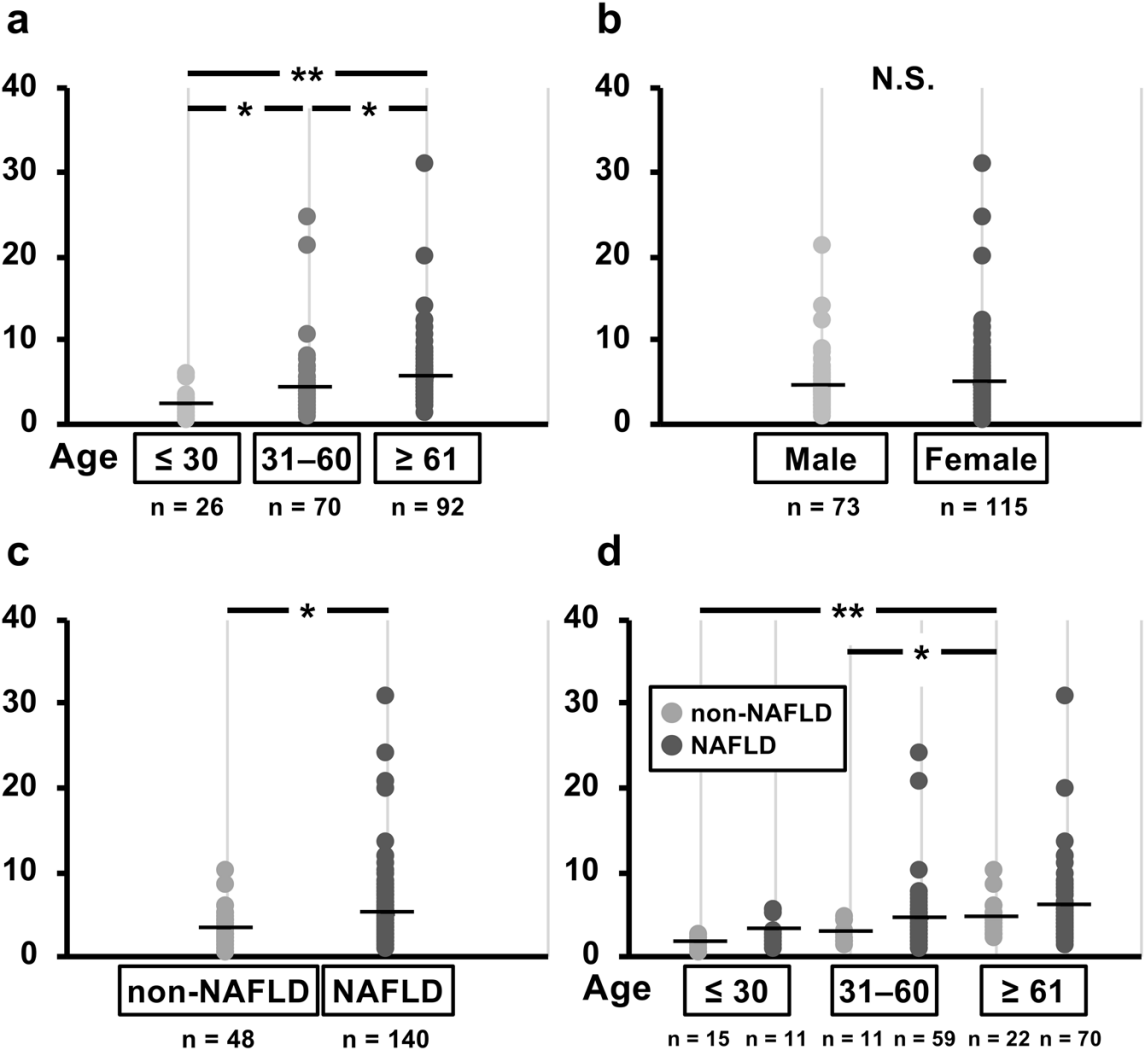
The comparisons of urinary titin-N fragment levels of the study subjects in younger, middle and elder age groups (**a**), the levels between males and females (**b**), the levels between NAFLD and non-NAFLD subjects (**c**), and the levels among NAFLD and non-NAFLD subjects in younger, middle and elder age groups. (**d**) The groups were compared using an ANCOVA with adjustment for gender and/or age as covariates. *P < 0.05, **P < 0.01; significantly different between the groups.

Urinary titin-N fragment levels were measured and compared between male and female subjects and showed no difference between the genders (Fig. 3). Comparison according to age group showed that urinary titin-N fragment levels were higher in subjects in the old-age group than in those in the young-age and middle-age groups. Urinary titin-N fragment levels were higher in NAFLD subjects than in non-NAFLD subjects.

Urinary titin-N fragment levels were compared between NAFLD and non-NAFLD subjects according to age groups, and they were found to increase with age in non-NAFLD subjects. Although urinary titin-N fragment levels tended to increase with age in NAFLD subjects, this increase was not statistically significant. Although these levels were higher in NAFLD subjects than in non-NAFLD subjects in the young-age group, they tended to be high in subjects in the middle-age and old-age groups.

**Figure 4:**
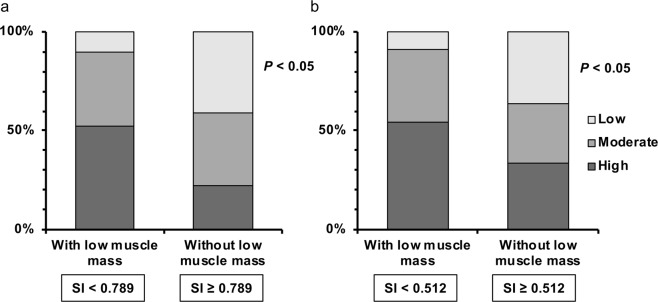
The comparisons of urinary titin-N fragment levels stratified by tertiles (low, intermediate, high) in the study subjects, with or without low muscle mass defined by sarcopenia index, SI (ref. ^24^). In both males (**a**) and females (**b**), the composition ratios of subjects with low, intermediate or high titin-N fragment levels were significantly different between those with and without low muscle mass.

All subjects with NAFLD were classified into the group with low muscle mass (SI < 0.789 for male, SI < 0.512 for female) and that without low muscle mass (SI ≥ 0.789 for male, SI ≥ 0.512 for female), and each group was divided into 3 subgroups according to urinary titin-N fragment levels: high, middle, and low (Fig. 4). Then, the distribution ratio of each of the 3 groups was compared. The ratio of subjects (both males and females) with high urinary titin-N fragment levels was significantly high in the group with low muscle mass, whereas in the group without low muscle mass, 22.2% of males and 33.7% of females were classified into the high urinary titin-N fragment level group.

When urinary titin-N fragment levels were compared with factors related to body composition, skeletal muscle, and the liver, they showed negative correlations with SMI (r = −0.166; *P* < 0.05), SI (r = −0.282; *P* < 0.001), SV ratio (r = −0.251; *P* < 0.001), skeletal muscle mass (r = −0.134; *P* < 0.05), grip strength (r = −0.203; *P* < 0.01), knee extension muscle strength (r = −0.191; *P* < 0.05), and DHEA levels (r = −0.348; *P* < 0.001). They showed positive correlations with muscle echo intensity (r = 0.361; *P* < 0.001), LSM (r = 0.177; *P* < 0.05), CAP (r = 0.162; *P* < 0.05), NFS (r = 0.455; *P* < 0.001), and FIB-4 index (r = 0.431; *P* < 0.001).

We performed multiple regression analysis with urinary titin-N fragment levels as the dependent variable and with narrowed down pathophysiological factors (except age) as independent factors that affected urinary titin-N fragment levels. On analysis (Table 3), 4 factors such as LSM, NFS, leg skeletal muscle mass, and SMI were identified as independent factors.

**Table 3:** Univariate and multivariate regression analysis for NAFLD pathophysiological factors associated with urinary levels of titin-N fragment for 133 study subjects

Parameter	Univariate analysis			
	B	Std. Error	Beta	P value
Liver stiffness measurement	0.094	0.038	0.177	<0.05
NAFLD fibrosis score	0.715	0.163	0.309	<0.001
FIB-4 index	0.679	0.209	0.234	<0.01
Skeletal muscle mass (total)	−0.100	0.045	−0.162	<0.05
Percent body fat	0.076	0.028	0.194	<0.01
Waist-to-hip-ratio	9.756	3.571	0.196	<0.01
Skeletal muscle mass (legs)	−0.432	0.158	−0.196	<0.01
Body fluid volume	−0.076	0.037	−0.149	<0.05
Lean body mass	−0.057	0.027	−0.152	<0.05
SMI	−0.129	0.056	−0.166	<0.05
SI	−0.129	0.056	−0.166	<0.05
SV ratio	−0.003	0.001	−0.179	<0.05
Muscle echo intensity	0.047	0.022	0.174	<0.05
Grip strength	−0.082	0.030	−0.202	<0.01
Albumin	−1.785	0.892	−0.147	<0.05
Platelet	−0.009	0.004	−0.155	<0.05
**Parameter**	**Multivariate analysis**			
NAFLD fibrosis score	0.640	0.169	0.295	<0.001
Liver stiffness measurement	0.167	0.055	0.253	<0.01
Skeletal muscle mass (legs)	−4.231	1.546	−2.052	<0.01
SMI	1.349	0.542	1.880	<0.05

SMI, skeletal muscle index; SI, sarcopenic index; SV ratio, skeletal muscle mass to visceral fat area ratio.

### Analysis of risk of liver fibrosis in study subjects in each titin-N fragment tertile

All study subjects were classified into the first (T1; high-level group), second (T2; intermediate-level group), and third (T3; low-level group) tertiles based on urinary titin-N fragment levels. We performed logistic regression analysis to calculate the relative risk of mild liver fibrosis and advanced liver fibrosis in each tertile using cut-off values of LSM (>7 pKa) and LSM (>10 pKa), respectively (Table 4). The relative risk was adjusted for cofounders (age and gender). When the risk of mild liver fibrosis in the T3 group was assumed to be 1, the relative risk of mild liver fibrosis significantly increased 1.77-fold in the T2 group and 2.81-fold in the T3 group. These results suggested that an increased urinary titin-N fragment level was a risk factor for the development of liver fibrosis.

**Table 4:** Unadjusted and adjusted Odds ratios with 95% confidence intervals of having liver fibrosis by tertiles of urinary titin-N fragment levels after adjusting for potential compounding factors (age, gender).

Tertiles of urinary titin-N fragment levels				
	3^rd^ Quartile	2^nd^ Quartile	1^st^ Quartile	*P* for trend
***Mild fibrosis (pKa* > *7)***				
Unadjusted	1	1.34 (0.58–3.08)	1.92 (0.86–4.30)	0.111
Model 1	1	1.76 (0.71–4.35)	2.81 (1.12–7.15)	0.030
Model 2	1	1.77(0.71–4.38)	2.81 (1.12–7.16)	0.030
***Advanced fibrosis (pKa* > *10)***				
Unadjusted	1	1.02 (0.36–2.91)	1.79 (0.68–4.67)	0.236
Model 1	1	1.25 (0.41–3.83)	2.38 (0.79–7.17)	0.124
Model 2	1	1.23 (0.40–3.79)	2.38 (0.79–7.16)	0.123

The 140 subjects with NAFLD (58 males and 82 females) with NAFLD and 48 non-NAFLD subjects (15 males and 33 females) were divided appropriately into the tertiles of urinary titin-N fragment levels.

Model 1: adjusted for age.

Model 2: adjusted for age and gender.

## Discussion

A summary of the results of this study is as follows: 1. Urinary levels of titin-N fragment increased with age, whereas no differences were found between male and female subjects in any age group. In the younger age group, NAFLD subjects had higher urinary levels than non-NAFLD subjects. 2. The urinary levels were negatively correlated with skeletal muscle mass and muscle strength (grip and knee extension strength). 3. The urinary levels were negatively correlated with both sarcopenia indices (e.g., SMI, SI) and sarcopenic obesity index (e.g., SV ratio). 4. The urinary levels were positively correlated with EMCL and muscle echo intensity of the rectus femoris muscle, which reflect muscle steatosis and atrophy as well as various parameters of liver fibrosis (LSM, NFS, FIB-4 index). 5. Multivariate analysis indicated that factors affecting the urinary levels of titin-N fragment t were LSM, NFS, leg skeletal muscle mass, and SMI. 6. In logistic regression analysis, NAFLD subjects with the high levels were likely to have an increased risk of liver fibrosis. Collectively, the urinary levels of titin-N fragment appeared to reflect a pathophysiology of muscle catabolic states in NAFLD, and could be used as biomarkers for prediction of the pathological conditions of sarcopenia and its related progression of liver fibrosis.

Subjects whose condition was diagnosed on the basis of measurement of walking speed, grip strength, and muscle mass according to the diagnostic criteria for sarcopenia^21^ have already been empirically shown to have sarcopenia, often with progression of associated hepatic lesions of NAFLD. Recently, it has been recognized that prevention of loss in muscle strength is particularly important in the management of sarcopenia. Thus, biomarkers that can assess the pathological conditions of sarcopenia or skeletal muscles associated with NAFLD at an early stage are needed.

To our knowledge, no prior report has investigated the urinary levels of titin-N fragment in NAFLD subjects. Interesting results were obtained when comparing the SI and the urinary levels. When subjects were classified into 3 groups (high, intermediate, low) according to the urinary levels, the percentage of subjects classified into the high-level group was high, as expected, in those who were diagnosed as being with low muscle mass on the basis of SI; however, some of those (22.2% in males and 33.9% in females) who were diagnosed as being without low muscle mass were classified into the high-level group (Fig. 4). The urinary levels were negatively correlated with grip strength, knee extension strength, and muscle mass, indicating that this parameter is a biomarker for earlier prediction of sarcopenia associated with NAFLD, compared to that using diagnosis according to the SI.

To predict sarcopenia associated with NAFLD, it is important to obtain information on not only muscle mass and strength of skeletal muscle but also muscle composition. The results of this study showed that the urinary levels of titin-N fragment were negatively correlated with muscle mass, which is a muscle index, and were positively correlated with muscle echo intensity, which reflects muscle steatosis, indicating that this parameter is a biomarker that can simultaneously predict quantitative and qualitative abnormalities of skeletal muscles in NAFLD subjects. The urinary levels were negatively correlated with grip strength and knee extension strength, which are anthropometric parameters, as well as the sarcopenic and sarcopenic obesity indices, indicating that this parameter can serve as a biomarker that can assess the presence of sarcopenia. In addition, the urinary levels were positively correlated with the NFS and FIB-4 index, which are parameters predictive of liver fibrosis. Thus, the urinary titin-N fragment levels should be clinically useful as a biomarker predictive of sarcopenia associated with progression of NAFLD.

Recently, ultrasonography has been utilized for noninvasive assessment of skeletal muscles. In this study, the echo intensity of skeletal muscles was found to be greater in NAFLD subjects than in non-NAFLD subjects in the same age group. Based on the results of multiple regression analysis, muscle thickness, SI, FPG, platelet count, and NFS were selected as independent factors affecting muscle echo intensity (data not shown). That is, in addition to decreased muscle mass and SI, progression of liver fibrosis and abnormal glucose tolerance were found to affect muscle echo intensity.

Although NAFLD subjects had increased muscle mass (Table 1), knee extension strength was decreased. Both EMCL and IMCL levels measured with ^1^H-MRS were positively correlated with muscle echo intensity (Fig. 1). The muscle echo intensity in NAFLD subjects may have increased because the apparent muscle mass is maintained while skeletal muscle fibers are decreased and/or fat is accumulated inside and outside the muscle fibers; therefore, muscle quality may have deteriorated.

Urinary levels of titin-N fragment were assessed in terms of muscle echo intensity, since an increase in muscle echo intensity might reflect the disease states of muscle atrophy; in fact, a positive correlation between these parameters was found. When tertile analysis was performed for muscle echo intensity, subjects with high echo intensity had higher urinary levels of titin-N fragment than those with low echo intensity (data not shown), whereas no differences in blood levels of myostatin, a myokine that suppresses muscle protein synthesis, were found between subjects with differences in muscle echo intensity.

The existence of an inter-organ network between skeletal muscles and the liver is becoming apparent. Sarcopenia is reportedly associated with progression of liver fibrosis in NAFLD subjects^9^. Our report showed that a low SV ratio is a contributor to progression of liver fibrosis^11,12^. Muscle echo intensity assessed in terms of parameters predictive of liver fibrosis (NFS, FIB-4 index) showed a positive correlation (data not shown). In the tertile analysis of muscle echo intensity, subjects with high echo intensity tended to have higher values for parameters predictive of liver fibrosis, compared with those in subjects with low echo intensity (data not shown).

The urinary levels of titin-N fragment were then assessed in terms of the disease state of liver fibrosis. It is of interest that independent factors affecting the urinary levels were found to be LSM, NFS, leg skeletal muscle mass, and SMI according to the results of multiple regression analysis (Table 3). Eventually, progression of liver fibrosis, in addition to sarcopenia, was found to be a factor that affects the urinary levels. Logistic analysis revealed that NAFLD subjects with high titin-N fragment levels were likely to have an increased risk of liver fibrosis (Table 4).

In conclusion, urinary levels of titin-N fragment reflect skeletal muscle deterioration and functional decline in NAFLD subjects. Moreover, increase in the titin-N fragment levels is associated with progression of liver fibrosis in NAFLD subjects. Urinary titin -N fragment is a useful biomarker for overall prediction of pathological conditions of skeletal muscles and the liver through liver-muscle crosstalk. In the context of liver-muscle crosstalk, sarcopenia may be a therapeutic target for chronic liver diseases including NAFLD; under such circumstances, measurement of titin-N fragment may be useful for the future interventional studies based on nutritional and exercise therapy.

## Methods

### Patients

This study was conducted on 253 outpatients (118 males and 135 females) enrolled between January 2017 and December 2018 that were followed for lifestyle-related liver diseases in our university hospital. Patients with at least 2 of the following ultrasonographic findings were selected as NAFLD subjects; “hepato-renal echo contrast,” “vascular blurring,” and “deep attenuation,” using the diagnostic criteria in evidence-based clinical practice guidelines for NAFLD^22^. Patients who did not have obesity, diabetes, or chronic liver disease and did not fulfill the abovementioned diagnostic criteria were selected as non-NAFLD subjects. The study protocol conformed to the ethical guidelines of the 1975 Declaration of Helsinki as reflected in prior approval by the Ethics Committee of Tsukuba University Hospital (H26–181). Written informed consent was obtained from each patient.

### Anthropometric measurements

Anthropometric measurements were performed as described previously^11^. The following sarcopenia-related indices were calculated: SMI (limb skeletal muscle mass [kg]/height [m]^2^)^23^; SI (limb skeletal muscle mass [kg]/BMI)^24^; and SV ratio^25^, a sarcopenic obesity index. Grip strength and knee extension strength were measured as parameters of physical ability as described previously^11^.

### Clinical and laboratory measurements

Blood for biochemistry was drawn from an elbow vein. Subjects fasted for 12 hours and refrained from vigorous exercise for 2 days before sampling, in principle. Separated serum was stored at −80 °C until measurement. Parameters related to liver function, glucose metabolism, and lipid metabolism were measured as previously described^26^. Surrogate markers, including HOMA-IR^27^, NFS^28^, and FIB-4 index^29^, were calculated from blood biochemistry data.

Commercially available ELISA kits were used to measure TBARS (Cayman Chemical, Ann Arbor, MI, USA) and hs-CRP (MBL Co., Ltd., Nagoya, Japan), which are inflammation/oxidative stress markers.

ELISA kits were also used to measure myostatin (Cusabio Biotech Co., Ltd., Wuhan, China), adiponectin (Sekisui Chemical Co., Ltd., Tokyo, Japan), IGF-1 (R & D Systems, Minneapolis, MN, USA), vitamin D (Cayman Chemical), DHEA (Enzo Life Science Inc., New York, NY, USA), and dihydrotestosterone (Abnova, Taipei City, Taiwan).

For spot urine measurement of titin-N fragment, a marker of muscle damages, subjects refrained from vigorous exercise for 1 week, in principle. Urine samples were stored at −80 °C until measurement. Titin-N fragment was measured with a commercially available ELISA kit (IBL Co., Ltd., Gunma, Japan)^19^. Measured values were corrected with urinary creatinine in consideration of concentrated or diluted urine as follows: titin-N fragment value (pmol/Cr) = measured titin-N fragment value (pmol/L) ÷ urinary Cr value (mg/dL).

### Skeletal muscle ultrasonography

Measurement was performed with an ultrasonic diagnostic imaging device (Aplio 500; Canon Medical Systems Corporation, Tokyo, Japan), using an 8–12-MHz electronic probe as previously described^30^. All images were taken once for each muscle, visually inspected, and analyzed by the same experienced operator. Image quality conditions were set using 90-dB gain, 7-cm depth, 9-MHz frequency, and 69-Hz dynamic range. The measurement site was the midpoint of a line connecting the anterior superior iliac spine of the dominant foot with the superior border of the patella in the resting supine position; the subcutaneous thickness over the thigh and the thickness, elasticity, and echo intensity of the rectus femoris muscle were measured.

For measurement of muscle echo intensity, longitudinal scanning images with ultrasound B-mode were visualized while focusing on the center of the muscle. The part excluding fascia was selected on the image, and the muscle brightness value was calculated with an 8-bit gray-scale. This is a method of quantifying the average value in the region as 256 tones, with 0 for black and 255 for white, in each pixel of the measurement region. Muscle elasticity was measured using Share Wave Elastography.

Ultrasound measurement of the rectus femoris muscle demonstrated high reliability for the thickness, echo intensity, and elasticity (Supplementary Table).

### Liver stiffness and steatosis

A FibroScan^®^502 device (Echosens, Paris, France) was used to measure these parameters. As previously described^11^, measurement was performed with a probe placed at a right intercostal space in the supine position under fasted conditions. The same skilled technician obtained both LSM and CAP. The M probe was used for a BMI < 30 and the XL probe was used for a BMI > 30. LSM was considered validated when the success rate was ≥60% and the interquartile range was <30%. The median of 10 validated measurements was adopted as the representative value.

### Proton magnetic resonance spectroscopy

A 3-Tesla MR device Philips Achieva (Philips Electronics Japan, Ltd., Tokyo, Japan) was used for ^1^H-MRS. As previously described^11^, the volume of interest was a single voxel and data were obtained using point-resolved spectroscopy. Data were analyzed with LC model (LCMODEL Inc., Oakville, ON, Canada) to calculate IMCL and EMCL.

### Statistical analysis

Statistical analysis was performed with SPSS Statistics 23.0 software (SPSS Inc., Chicago, IL, USA). Each measured value of subjects was expressed as mean ± standard error (mean ± SE). Subjects were classified into 3 groups by age (≤30 years, 31 to 60 years, ≥61 years); each pathophysiological factor of NAFLD among these 3 groups was compared using ANCOVA with adjustment for gender as a covariate. The intergroup comparison was made using Bonferroni’s multiple comparison test. Frequency of each disease and diseased conditions was compared using Pearson’s χ^2^ test. Univariate and multivariate regression analyses were performed for associations between urinary levels of titin-N fragment and each NAFLD pathophysiological factor in the subjects. Odds ratios (ORs) were obtained from logistic regression analysis, and the results were presented as ORs with a 95% confidence interval (CI). A *P*-value less than 5% was considered statistically significant.

## Supplementary information

Supplemental Table
