# Supplementary material for: Urinary Levels of Titin-N Fragment, a Skeletal Muscle Damage Marker, are Increased in Subjects with Nonalcoholic Fatty Liver Disease
Source: Sci Rep. 2019 Dec 20;9:19498. doi: 10.1038/s41598-019-56121-7 (PMC6925124; doi:10.1038/s41598-019-56121-7)
Supplement: Supplementary file 1 — Supplemental Table [file 41598_2019_56121_MOESM1_ESM.docx]

| Table: The means and ranges of coefficients of variation (CVs) in measurement of thickness, echo intensity, and elasticity of rectus femoris muscle. | | | | | | | | | | | | |
| --- | --- | --- | --- | --- | --- | --- | --- | --- | --- | --- | --- | --- |
|  | Muscle  thickness  (mm) | | | CV | Echo  intensity | | | CV | Muscle  elasticity  (m/s) | | | CV |
| subject 1 | 15.67 | ± | 0.17 | 0.011 | 102.08 | ± | 0.47 | 0.005 | 2.02 | ± | 0.08 | 0.040 |
| subject 2 | 14.73 | ± | 0.41 | 0.011 | 99.18 | ± | 1.21 | 0.012 | 2.39 | ± | 0.04 | 0.019 |
| subject 3 | 11.6 | ± | 0.43 | 0.037 | 100.66 | ± | 1.19 | 0.012 | 2.24 | ± | 0.08 | 0.035 |
| subject 4 | 13.6 | ± | 0.24 | 0.018 | 102.75 | ± | 0.41 | 0.004 | 1.87 | ± | 0.06 | 0.033 |
| subject 5 | 14.03 | ± | 0.45 | 0.032 | 101.50 | ± | 1.26 | 0.012 | 1.82 | ± | 0.07 | 0.036 |
| subject 6 | 16.77 | ± | 0.26 | 0.016 | 85.27 | ± | 1.22 | 0.014 | 1.78 | ± | 0.03 | 0.019 |
| subject 7 | 19.1 | ± | 0.16 | 0.009 | 86.91 | ± | 0.89 | 0.010 | 1.88 | ± | 0.04 | 0.020 |
| mean |  |  |  | 0.021 |  |  |  | 0.010 |  |  |  | 0.029 |
| Thickness, echo intensity, and elasticity of the muscle were measured in 7 subjects three times on the same day to study the reproducibility. | | | | | | | | | | | | |
